# Prevalence of myalgic encephalomyelitis/chronic fatigue syndrome (ME/CFS) in three regions of England: a repeated cross-sectional study in primary care

**DOI:** 10.1186/1741-7015-9-91

**Published:** 2011-07-28

**Authors:** Luis C Nacul, Eliana M Lacerda, Derek Pheby, Peter Campion, Mariam Molokhia, Shagufta Fayyaz, Jose CDC Leite, Fiona Poland, Amanda Howe, Maria L Drachler

**Affiliations:** 1 Department of Nutrition and Public Health Interventions Research, London School of Hygiene and Tropical Medicine (LSHTM), Keppel Street, London, WC1E 7HT, UK; 2 Foundation for Genomics and Population Health (PHGF), Cambridge, CB1 8RN, UK; 3 National ME Observatory Project Coordinator, and Bucks New University, Queen Alexandra Road, High Wycombe, Buckinghamshire, HP11 2JZ, UK; 4 Hull-York Medical School, University of Hull, Hull, HU6 7RX, UK; 5 Department of Primary Care and Public Health Sciences, Division of Health and Social Care Research. Kings College, London, SE1 3QD, UK; 6 University of East Anglia, Norwich, NR47TJ, UK

## Abstract

**Background:**

Myalgic encephalomyelitis/chronic fatigue syndrome (ME/CFS) or chronic fatigue syndrome (CFS) has been used to name a range of chronic conditions characterized by extreme fatigue and other disabling symptoms. Attempts to estimate the burden of disease have been limited by selection bias, and by lack of diagnostic biomarkers and of agreed reproducible case definitions. We estimated the prevalence and incidence of ME/CFS in three regions in England, and discussed the implications of frequency statistics and the use of different case definitions for health and social care planning and for research.

**Methods:**

We compared the clinical presentation, prevalence and incidence of ME/CFS based on a sample of 143,000 individuals aged 18 to 64 years, covered by primary care services in three regions of England. Case ascertainment involved: 1) electronic search for chronic fatigue cases; 2) direct questioning of general practitioners (GPs) on cases not previously identified by the search; and 3) clinical review of identified cases according to CDC-1994, Canadian and Epidemiological Case (ECD) Definitions. This enabled the identification of cases with high validity.

**Results:**

The estimated minimum prevalence rate of ME/CFS was 0.2% for cases meeting any of the study case definitions, 0.19% for the CDC-1994 definition, 0.11% for the Canadian definition and 0.03% for the ECD. The overall estimated minimal yearly incidence was 0.015%. The highest rates were found in London and the lowest in East Yorkshire. All but one of the cases conforming to the Canadian criteria also met the CDC-1994 criteria, however presented higher prevalence and severity of symptoms.

**Conclusions:**

ME/CFS is not uncommon in England and represents a significant burden to patients and society. The number of people with chronic fatigue who do not meet specific criteria for ME/CFS is higher still. Both groups have high levels of need for service provision, including health and social care. We suggest combining the use of both the CDC-1994 and Canadian criteria for ascertainment of ME/CFS cases, alongside careful clinical phenotyping of study participants. This combination if used systematically will enable international comparisons, minimization of bias, and the identification and investigation of distinct sub-groups of patients with possibly distinct aetiologies and pathophysiologies, standing a better chance of translation into effective specific treatments.

## Background

Despite being described more than five decades ago [1,2], myalgic encephalomyelitis/chronic fatigue syndrome (ME/CFS) remains controversial and poorly understood by health professionals [3] and the wider public. ME/CFS diagnosis is still one of exclusion and is mainly based on the presence of persistent or recurrent chronic fatigue associated with a range of symptoms, which often fluctuate, sometimes even over short periods of time. A number of case definitions and clinical labels, with varying degrees of sensitivity and specificity [4,5] have been used to characterize the cases [2,6-13].

Primary care practitioners have used diverse case definitions and diagnostic labels to describe their chronically fatigued patients, for example CFS, ME, post viral fatigue syndrome, fibromyalgia, asthenia, and post-viral debility [14]. Terminological variations and inconsistencies in how definitions and labels are used have rendered studies on the occurrence and distribution of ME/CFS in populations difficult to compare, unless robust and consistent methods, including case definitions that can discriminate ME/CFS cases from cases of chronically fatigued non-ME/CFS, are applied. Precise statistics about the burden of the disease, including its spatial distribution and level of severity, are crucial for adequate planning of health and social services and of research. Our approach to the selection of case definitions applied in the study reported in this paper is described in the Methods section below. While both ME/CFS and chronically fatigued non-ME/CFS patients have debilitating conditions [4,5] and need high levels of health care and social support, those with ME/CFS may have distinct needs in relation to diagnosis and therapeutic options, as they are likely to constitute a distinctive nosological condition, albeit that the pathophysiology is still unclear. These are still largely unmet for reasons likely to be related to their perceived lack of recognition as having a legitimate condition. Moreover, the absence of a definitive diagnosis may lead to their receiving inadequate care and support [15].

This paper describes the prevalence rates and annual incidence risk of ME/CFS in primary care in parts of England, based on samples from London, East Anglia and East Yorkshire, and discusses the implications of the frequency statistics and the use of different case definitions for planning health and social care and research.

## Methods

The study was part of the ME/CFS Observatory, a collaborative research program that encompasses quantitative and qualitative studies and the piloting of an ME/CFS specific disease register [16,17]. It was carried out following approval from the Multi-Centre Research Ethics Committee in London (London-MREC - 06/MRE/02/57), the London School of Hygiene and Tropical Medicine Ethics Committee and the local NHS Research Governance Units in London, East Anglia and East Yorkshire.

The study comprised two cross-sectional surveys twelve months apart, involving a population of 143,000 people between 18 to 64 years of age from 29 general practices in three regions of the country, carried out between 2007 and 2010. Five of the general practices were located in parts of London, five in East Anglia and 19 in East Yorkshire. Some patients were also recruited from a specialist ME/CFS service in East Anglia, run by GPs with special interest in ME/CFS, and data from these patients are included in some of the analyses, but excluding all analyses leading to prevalence and incidence estimates. The inclusion of 45 cases from the specialist clinic aimed to add cases, and thus power to some of the epidemiological analyses. Identical procedures for diagnosis were used in GP practices and specialist clinics, except that the latter contains a further level of diagnostic confirmation, as cases are referred by GPs and receive diagnostic confirmation by another health professional (and we compared the results with and without these patients). The study areas were selected within the geographical areas of the participating institutions, and to capture populations with large representation from ethnic minorities (London), with Caucasians over-represented in Norfolk and East Yorkshire, both of which had proportionately large rural populations. We were careful to choose practices with GPs with experience in diagnosing ME/CFS. The effect of this approach on representativeness of cases was judged to be justified, otherwise we would have severely under-estimated the true disease occurrence, as GPs without an interest in ME/CFS would more often fail diagnose it. This resulted in an imbalance in numbers of GP practices in the three -regions, with East Yorkshire over-represented. The sample size was calculated (using EPI-INFO version 3.7 software) to detect a prevalence of ME/CFS of 0.5% with a confidence level of 95% and precision of 0.4%. Lower precision would be achievable for population subgroups defined by geographic location and gender, and for incidence risk estimation.

**Figure 1:**
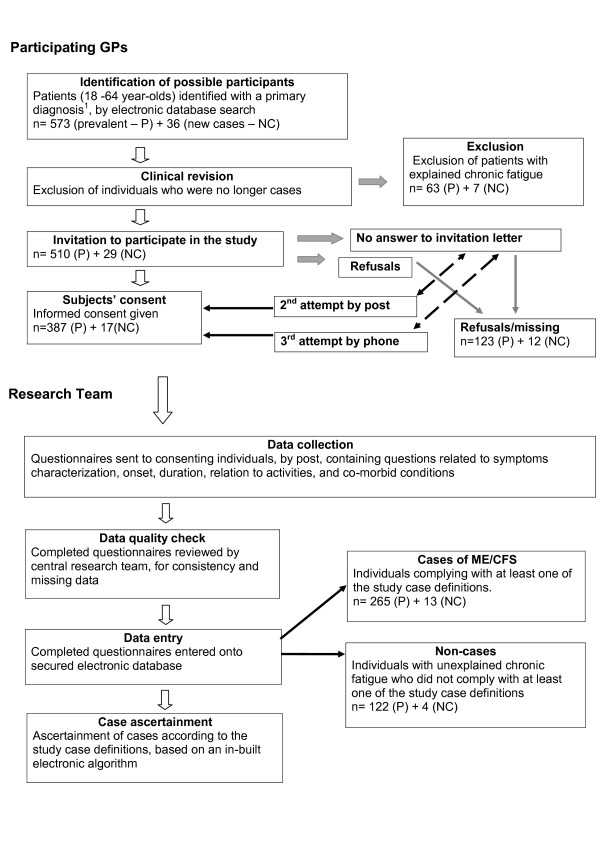
**Flowchart of recruitment and ascertainment of cases**. Note: ^1 ^Chronic fatigue syndrome (CFS), myalgic enchephalopathy (ME), post viral asthenic syndrome (PVAS), fatigue syndrome (FS), fibromyalgia (FMS), post-infectious encephalitis (PIE), and any other codes offered by the GPs to denote cases of ME/CFS.

Recruitment of patients followed a staged approach for the identification of cases (Figure 1), starting with a search of general practitioners' (GP) electronic databases. The participating general practices identified and selected all 18-to-64-year-old patients whose medical records contained any of the following diagnoses: CFS, ME, post-viral asthenic syndrome (PVAS), fatigue syndrome (FS), fibromyalgia (FMS), post-infectious encephalitis (PIE), and any other codes offered by the participating GPs to denote cases of ME/CFS. The use of the above terminologies by GPs was not based on distinct case definitions, but on diagnostic terms occurring in the READ clinical lexicon, which is the basis for clinical coding and classification in GP computer systems in the UK. The READ lexicon does not contain definitions, but simply reflects practices by individual GPs in relation to naming cases with similar clinical pictures [18]. The terms listed above we refer to as primary diagnoses. The GPs were asked to review all cases identified with primary diagnoses, using the study case definitions, including the presence of symptoms for more than six months, and to exclude those they no longer considered as cases, either because of clinical improvement or the finding of any other clinical condition explaining their symptoms. In addition they were asked to identify by recall any further cases that might not have been picked up by the electronic searches.

The GPs then sent a letter to the selected patients inviting them to participate in the study. Potential participants received an information pack containing the invitation letter from their GP, an information sheet about the study, consent forms and a research study questionnaire. The questionnaire developed by the authors was a symptom checklist containing questions on symptoms and functional capacity enabling case ascertainment and classification according to one or more of the diagnostic criteria used in the study. Those with ME/CFS diagnosed by the GPs but who did not meet any of the study criteria were considered as non-cases for the purpose of this study.

For someone to be confirmed as a case of ME/CFS for the purpose of the study, compliance with any of three distinct case definitions was required. These were the Centers for Disease Control (CDC) definition of 1994, referred to here as the CDC-1994 criteria [9]; the clinical working case definition established in Canada by an Expert Medical Consensus Panel, known as the Canadian criteria [7], which appears more stringent than the CDC-1994 definition, and to define as having ME/CFS a more unequivocally ill group of patients. We also used a case definition developed by two of the authors so as to investigate its usefulness for epidemiological research, and to attempt to validate it. It is referred to in this report as the Epidemiological Case Definition - ECD [11]. This was developed by requesting GPs to complete a symptom checklist with respect to all patients on their practice lists with unexplained chronic fatigue. The symptom checklist comprised all the clinical features in the various case definitions of ME/CFS, including the CDC-1994 and Canadian definitions. Patients were then assigned, following perusal of the completed checklists by an expert reference group, to disease or non-disease groups. Discriminant analysis was then applied to identify which symptoms were most effective in assigning cases accurately to the disease or non-disease groups. The symptoms thus identified became *de facto *the epidemiological case definition.

Compliance with the above case definitions was ascertained by use of a computerized algorithm which mapped symptom profiles onto the case definitions. Estimates of the prevalence of ME/CFS were then made on the basis of cases identified in this way, using the number of patients in the practice lists as denominators. The choice of different diagnostic criteria was intended to enable international comparisons, and to examine distinct patterns of symptoms and severity. The CDC-1994 and Canadian criteria in particular were chosen as they have been widely used worldwide.

For their further characterization, all participants were also asked to complete a visual analogue pain scale [19] and a scoring instrument to access the severity of the fatigue symptom based on the Karnofsky rating scale [20]. For the pain scale, the respondents marked their level of pain on a 10 cm line ranging from 0 = no pain to 10 = pain as bad as possible, the measured points in the line represented the pain scores (as a visual analogue). The severity of fatigue was assessed through a scale ranging from 0 - no symptoms with exercise, normal overall activity to 100 - severe symptoms on a continuous basis, bed ridden constantly, unable to care for self.

After one year (study phase 2), a new electronic search was carried out in the same GP practices to identify any patients with a new diagnosis of ME/CFS since first recruitment, and to enable the estimation of annual incidence risks. Procedures similar to those described above were followed to identify and contact patients and to confirm diagnosis.

We used the software Stata/IC 11.1 for Windows to undertake the analysis, and tested statistical significance with Chi-squared or Fisher's exact tests for comparing categorical variables [21]. Continuous variables were compared using the Student t-test or Wilcoxon rank sum test [21], where appropriate. Crude annual incidence risks and prevalence rates were calculated with the number of people aged 18 to 64 years registered with each of the general practices as the denominators. On the basis of these, estimates were made for adjusted regional incidence risks and prevalence rates (and 95% confidence intervals) according to each and any of the case study definitions, using the direct standardization method [21]. Population estimates for England for mid-2008 (Office for National Statistics) were used as the reference population [22]. The age standardised incidence risks and prevalence rates were then adjusted by response rate in each region for both genders, using the assumption that the occurrence of ME/CFS did not differ between respondents and non-respondents.

We compared the clinical presentation (report of symptoms) of patients meeting each study case definition and non-cases, that is the chronically fatigued patients recruited by GPs that did not conform to any of the adopted ME/CFS diagnostic criteria. To remove duplications when comparing the proportion of the various symptoms in patients meeting different case definitions, we re-grouped cases into two groups, those conforming both to CDC-1994 and Canadian criteria (the Canadian group), and those conforming to CDC-1994 only. Patients conforming only to the ECD were excluded from both groups of patients. This decision was based on the observation that nearly all patients meeting the Canadian criteria also met the CDC-1994 criteria.

## Results

### Characterization of study population

Our sample population was selected from three areas, which were quite distinct, but which covered a broad range of characteristics. These included East Anglia, a largely rural area with numerous small to medium-sized market towns and few large population centres; East Yorkshire, with one large city manifesting many of the features of industrial decline and inner city deprivation and a large rural hinterland; and London, with a large non-indigenous and highly mobile population, and marked economic polarization, with very affluent and significantly deprived populations living in close proximity [23]. The practices in London come from areas where the proportion of people from Black and Ethnic Minorities (BEM) is around 40%, with up to a third born outside the UK, large numbers of whom were from south Asia and sub-Saharan Africa [24]. The proportion of individuals under five years of age was in most places between 6% and 6.5%, while the proportion of those aged over 65 years varied between 7% and 15%. Between 20% and 25% of the adults had no formal qualifications [23].

By contrast, the practices in Norfolk were from an area where the proportion of people from BEM is less than 3%, and less than 5% were born outside the UK [24]. The proportion of individuals under five years of age was 5%, and of those over 65 was 20%; 32% of the adult population had no formal qualifications [23]. East Yorkshire has a non-white population of 7%, with 19% over the age of 65 years. This region tended to have lower life expectancy and educational achievement than the other two [23].

We covered populations ranging from some of the poorest in the country to some of the most affluent. For example, central Hull, in East Yorkshire, has the 10^th ^worse Index of Multiple Deprivation (IMD) in England (out of 152 areas), while others in London have IMDs ranking from 19^th ^to 118^th^, and Norfolk ranks 108^th ^in the country. In contrast, rural areas in East Yorkshire are relatively well-off with mean IMD rank of 135^th ^[25]. Some demographic characteristics of the study population are presented in Table 1.

**Table 1:** Demographic data on GP practices and study population in the three studied regions

Region	Regional Population^a^						GP practices' population^b ^		GP Practices in the region^c^ (n)	GPs in the region^c^ (n)	Participating practices (n) (%)		Participating GPs (n) (%)	
									
	Total (n)	18-64 year-olds (%)	Gender		Ethnicity									
									
			Male (%)	Female (%)	White (%)	BEM^d ^(%)	18-64 year-olds (n)	18-64 year-olds (%)						
East Yorkshire	597,200	60.4	48.8	51.2	98.3	1.7	82,080	22.8	105	375	19	18.1	162	43.2
London^e^	508,400	65.1	48.6	51.7	61.1	38.9	33,047	10	86	414	5	5.8	31	7.5
East Anglia	964,800	59.3	48.6	51.4	98.5	1.5	28,026	4.9	136	672	5	3.7	26	3.9

^a^Estimated population mid-2008. Source: Office for National Statistics - http://www.statistics.gov.uk/statbase/product.asp?vlnk = 15106; ^b^Source: http://www.nhs.uk/Services/Pages/AcuteTrustList.aspx?trustType=PrimaryCare; ^c^Source: https://www.nhs.uk/servicedirectories/Pages/ServiceSearch.aspx; ^d^BEM: Black and Ethnic Minorities; ^e^Harrow and Southwark Primary Care Trust areas.

### Baseline recruitment

From the 573 patients identified as having any primary diagnoses (in their medical records), 63 (11%) were excluded as they no longer had chronic fatigue at the time of the search, or had been diagnosed with other conditions explaining their symptoms, such as hypothyroidism, hyperparathyroidism, hydrocephaly, cancer, psychiatric conditions, Crohn's disease and rheumatoid arthritis. The proportion of individuals included with primary diagnoses was evenly distributed among the three geographical regions: 34.1% from London, 32.1% from East Anglia and 33.7% from East Yorkshire. More than 70% (394/510) were women. The median age was 49.3 years (interquartile range (IR) = 40.7 to 56.1) for all cases. For men it was 49.1 years (IR = 39.7 to 55.4) and for women 47.5 years (IR = 38.5 to 55.4); p = 0.56). The response rate was 75.9% (387/510).

### Second recruitment (12 months following initial recruitment)

One year after the baseline recruitment, an additional 36 new cases were identified from the GP records. Seven were excluded by the GPs, and 29 were invited to take part in the study. Seventeen of them responded (response rate of 58.6%), of whom 13 (76.5%) met at least one of the study case definitions for ME/CFS.

### ME/CFS caseness for prevalent and incident cases

Two hundred and seventy eight of the recruited patients (68.8%) met at least one of the ME/CFS study case definitions, while 122 were considered non-cases. Of the included cases, 95.3% (265) were prevalent and 4.7% (13) incident cases, that is identified at the baseline and at the 12 months recruitment, respectively. We observed that GPs' diagnoses of CFS and FMS were more likely to comply with the study case definitions (positive predictive values (PPV) = 72.3% and 71.1%, respectively), than less specific labels such as fatigue syndrome. All labels referring to post-viral syndromes showed PPV lower than 50% (Table 2).

**Table 2:** Positive predictive value of primary diagnoses given by GPs for ME/CFS

GP's primary diagnoses	ME/CFS		Not ME/CFS			Positive Predictive Value (%)
			
	n	%	n	%	*Total*	
Any primary diagnosis	278	100.0	126	100.0	404	68.8
Chronic fatigue syndrome	175	62.9	67	53.2	242	72.3
Fibromyalgia syndrome	81	29.1	33	26.2	114	71.1
Fatigue syndrome	3	1.1	2	1.6	5	60.0
Post-viral asthenic syndrome	16	5.8	18	14.3	34	47.1
Other ^a^	3	1.1	6	4.8	9	33.3

^a^That is, post-viral debility, post-viral syndrome, post-viral fatigue

The majority of ME/CFS cases complied with the CDC-1994 criteria (97.1%), 52.5% conformed to the Canadian criteria and 16.9% to the ECD. However, there was a considerable overlap among case definitions, as shown in Table 3. For example, 270 patients met the CDC-1994 criteria. Of these, 127 also met the Canadian criteria, while 18 met both the Canadian and ECD definitions. A total of 103 fulfilled the CDC-1994 definition but neither of the others.

**Table 3:** Distribution of ME/CFS cases (prevalent and incident) according to the study case definitions

ME/CFS Diagnostic Criteria		n	%
Any ME/CFS diagnostic criteria		278	100.0
CDC - 1994		270	97.1
CDC - 1994 only^a^		103	37.1
Canadian		146	52.5
Canadian only^a^		1	0.4
ECD		47	16.9
ECD only^a^		7	2.5
Overlap of case definitions	CDC - 1994 and Canadian	127	45.7
	CDC - 1994, Canadian and ECD	18	6.5
	CDC - 1994 and ECD	22	7.9
			

^a^Non compliant with other definitions

### Prevalence estimates

When the three case definitions used in the study were considered concurrently, we found an overall ME/CFS prevalence rate of 0.20%, after correction for non-response. The rate varied slightly among and within the regions, with London presenting the highest rate of 0.31% (ranging from 0.12 to 0.50%), followed by East Anglia of 0.25% (ranging from 0.15 to 0.33%), and East Yorkshire at 0.14% (ranging from 0.03 to 0.32%). These rates also varied by gender, with the overall and within the regions prevalence rates consistently higher in women than in men (Table 4). The highest prevalence rate was found among women in London (0.48%); while in men, the highest prevalence rate was found in East Anglia (0.15%). The prevalence rate of cases meeting the CDC-1994 definition was 0.19%, of cases meeting the Canadian definition 0.11%, and of cases meeting the ECD 0.03%.

**Table 4:** Crude, directly standardised and adjusted prevalence rates of ME/CFS

GP Practices	ME/CFS cases (n)	Population 18 - 64 years old	Crude prevalence rates (%)			Directly standardised prevalence rates^a^ (%)						Prevalence rates adjusted by response rates^b^ (%)		
	
	T	T	T	M	F	T	(95% CI)	M	(95% CI)	F	(95% CI)	T	M	F
All practices	219	143,153	0.15	0.06	0.25	0.15	0.13 - 0.17	0.07	0.05 - 0.09	0.23	0.20 - 0.26	0.20	0.09	0.30
London	32	33,047	0.10	0.02	0.18	0.10	0.07 - 0.14	0.02	0.01 - 0.06	0.18	0.12 - 0.26	0.31	0.13	0.48
East Anglia	71	28,026	0.25	0.10	0.41	0.24	0.19 - 0.30	0.11	0.06 - 0.19	0.36	0.27 - 0.46	0.25	0.15	0.36
East Yorkshire	116	82,080	0.14	0.06	0.23	0.14	0.11 - 0.17	0.06	0.04 - 0.10	0.20	0.16 - 0.25	0.14	0.05	0.20

^a^Reference population: England mid-2008 population (source: Office for National Statistics - http://www.statistics.gov.uk/statbase/product.asp?vlnk = 15106); ^b^Assumption: The respondents and non-respondents have proportionally the same number of ME/CFS cases; T, Total; M, male; F, female; based on 29 GP clinics

### One year incidence risk

The overall risk was 0.15‰. It was 0.33‰ (range 0 to 1.07‰) in London, 0.24‰ (range 0.20 to 0.26‰) in East Anglia, and 0.02‰ (range 0.13 to 0.35‰) in East Yorkshire. The overall risk was higher in women than in men (respectively, 0.41‰ and 0.06‰). Annual incidence risks also differed among and within the regions (Table 5).

**Table 5:** Crude, directly standardized, and adjusted annual incidence risk of ME/CFS

GP Practices	ME/CFS cases (n)	Population 18 - 64 years old	Crude annual incidence risk (‰)			Standardized annual incidence risk^a^ (‰)						Annual incidence risk corrected response rates^b ^(‰)		
			
			T	M	F	T	(95% CI)	M	(95% CI)	F	(95% CI)	T	M	F
All practices	13	143,153	0.09	0.04	0.14	0.09	0.05 - 0.15	0.04	0.01 - 0.11	0.13	0.06 - 0.24	0.15	0.06	0.41
London	4	33,047	0.12	0.12	0.13	0.12	0.03 - 0.31	0.10	0.01 - 0.37	0.12	0.01 - 0.43	0.33	0.27	0.31
East Anglia	7	28,026	0.25	0.07	0.44	0.24	0.10 - 0.50	0.07	0 - 0.39	0.38	0.14 - 0.83	0.24	0.07	0.38
East Yorkshire	2	82,080	0.02		0.05	0.02	0 - 0.09			0.05	0.01 - 0.16	0.02		0.05

^a^Reference population: England mid-2008 population (source: Office for National Statistics - http://www.statistics.gov.uk/statbase/product.asp?vlnk = 15106);

^b^Assumption: The respondents and non-respondents have proportionally the same number of ME/CFS cases; T, Total; M, male; F, female; based on 29 GP clinics.

### Characterization of prevalent cases

One hundred and seventy five prevalent case individuals completed the second questionnaire (response rate = 66%). More than half of these cases were between 45 and 65 years of age (66.9%). Men were slightly older than women (median age 53.2 (IR = 47.5-60.2) and 49.6 (IR = 40.4 -56.5), respectively (*P *= 0.05). Only 7.5% (13/174) considered themselves as not White British, and almost half completed secondary education (91/172). Overall, 74.6% (129/173) were married or living with a partner.

### Symptoms and case definitions

The onset of symptoms was reported to range from 6 months to 27 years before recruitment to the study. The median time from the start of symptoms was 8 years (IR = 4 to 15). One hundred and thirty-three (76%) respondents recognized triggering factors for their symptoms, such as episodes of infection (57.9%, of which 74% were reported as viral infections), psychological stress (24.8%), trauma or surgery (11.3%), and other factors (6.0%). We found no differences in the distribution of triggering factors between the cases in conformity with the Canadian/CDC-1994 criteria and those in conformity with the CDC-1994 criteria only.

The frequency of symptoms (assessed in all 265 prevalent cases) varied according to case definitions used. However, unrefreshing sleep appeared across the case definitions as the most frequent (proportions ranged from 97% to 100%), followed by memory and concentration problems (from 95% to 98%), joint (76% to 91%) and muscle pain (74% to 95%). In general, the frequency of each of the symptoms investigated was highest in those complying with the Canadian criteria, and lowest in those conforming to the ECD (Table 6). Table 7 shows that the proportion of patients reporting sleep dysfunction and symptoms related to neurological/cognitive, immunological and psychological functions was significantly higher in the group conforming to the Canadian criteria than in the group conforming only to the CDC-1994 criteria. The proportions were similar only in relation to pain in two or more joints without inflammatory signs (*P *= 0.29). Most, but not all of the reported symptoms were proportionally higher in the group conforming to the CDC-1994 criteria only (non-Canadian) than in the non-cases group. In the group complying with the Canadian criteria all the symptoms were proportionally higher than in the non-cases, these differences being statistically significant. Comparisons between the groups conforming to the Canadian criteria and CDC-1994 criteria only (non-Canadian) have shown that all the reported symptoms were higher in the Canadian group (*P *= 0.000, apart for migraine where *P *= 0.002, these *P *values are not presented in Table 7).

**Table 6:** Proportion of reported symptoms in prevalent cases, according to study case definitions^a^

Current symptoms		All cases (n = 265)	CDC-1994 (n = 258)	Canadian (n = 142)	ECD (n = 43)
Sleep dysfunction	Unrefreshing sleep	97.4	97.3	100.0	97.7
	Sleep problems	84.9	85.3	94.4	73.0
					
Neurological/Cognitive manifestations	Memory/concentration problems	94.3	95.3	98.0	95.3
	Difficulty thinking	81.5	82.0	93.0	74.4
	Unexplained muscle weakness	75.1	74.8	88.0	70.0
	Light/noise sensitivity	65.7	66.0	82.4	65.1
	Difficulty in understanding things	58.1	59.3	76.1	48.8
	Confusion or disorientation	54.7	55.0	78.2	44.2
					
Pain	Pain in two or more joints	89.8	91.1	94.4	76.7
	Muscle pain	88.9	89.5	95.1	74.4
	Muscle discomfort	86.8	88.0	94.4	76.7
	Migratory joint pain	75.8	76.4	85.2	53.5
	Headaches	64.5	65.5	74.6	60.5
	Migraine	28.3	28.3	36.6	25.6
					
Fatigue/tiredness	Fatigue after exercise (> 24 hours)	81.1	80.6	100.0	90.7
	Intolerance to exercise	71.7	71.7	85.2	81.4
	Malaise after exertion (> 24 hours)	69.1	69.4	85.9	86.0
					
Psychological	Anxiety	70.9	72.5	83.1	39.5
	Depression	55.8	56.2	67.6	11.6
					
Autonomic	Sweatiness/cold hands and feet	66.4	67.4	88.0	55.8
	Intolerance to be on your feet	61.1	60.8	79.6	55.8
					
Immunological	Sore throat	57.0	56.7	71.8	60.5
	Tender glands in the neck/armpits	57.0	57.7	74.6	55.8
	Mild fever or chills	52.8	52.7	75.3	46.5

^a^All cases complying with each of the definitions are shown in the columns; therefore cases meeting more than one diagnostic criterion appear more than once.

**Table 7:** Reported symptoms in ME/CFS prevalent cases according to compliance with Canadian criteria and non-cases^a^

Current symptoms		Compliance with Canadian criteria		
		No (n = 97) (%)	Yes (n = 125) (%)	Non-case (n = 122) (%)
Sleep dysfunction	Unrefreshing sleep	93.8^d^	100.0^b^	63.1
	Sleep problems	74.2^b^	97.6 ^d^	56.6
				
Neurological/Cognitive manifestations	Memory/concentration problems	89.7^d^	97.6^b^	54.1
	Difficulty thinking	69.1^d^	93.6 ^d^	42.6
	Unexplained muscle weakness	60.8^c^	88.0 ^d^	41.0
	Light/noise sensitivity	44.3^b^	82.4 ^d^	28.7
	Difficulty in understanding things	37.1	77.6 ^d^	32.0
	Confusion or disorientation	28.9	78.4 ^d^	26.2
				
Pain	Pain in two or more joints	89.7^d^	94.4 ^d^	46.7
	Muscle pain	86.6^d^	95.2 ^b^	48.4
	Muscle discomfort	82.5^d^	93.6 ^b^	48.4
	Migratory joint pain	71.1^d^	87.2 ^c^	40.2
	Headaches	51.5^c^	76.0 ^d^	32.8
	Migraine	15.5	39.2 ^d^	21.3
				
Fatigue/tiredness	Fatigue after exercise (> 24 hours)	52.6^b^	100.0 ^d^	37.0
	Intolerance to exercise	50.5^b^	84.8 ^d^	34.4
	Malaise after exertion (> 24 hours)	41.2^b^	85.6 ^d^	26.2
				
Psychological	Anxiety	63.0	88.0 ^d^	56.6
	Depression	50.5	75.2 ^d^	41.0
				
Autonomic	Sweatiness/cold hands and feet	42.3	88.8 ^d^	38.5
	Intolerance to be on your feet	38.1^b^	80.8^d^	24.6
				
Immunological	Sore throat	37.1	71.2 ^d^	27.0
	Tender glands in the neck/armpits	36.1	73.6 ^d^	27.9
	Mild fever or chills	23.7	77.6 ^d^	29.5

^a^All cases met the CDC-1994 criteria; *P*-values are shown for comparisons of proportions of symptoms between cases that do not conform to the Canadian criteria and non-cases; and between cases that conform to Canadian criteria and non-cases. The following symbols show respective *P*-values: ^b^P < 0.05; ^c^*P *< 0.005; ^d^*P*≤0.001; the results comparing ECD with other diagnostic criteria and non-cases are not presented due to small numbers.

Fatigue was predominantly reported as moderate to severe, with median of 60 (IR = 50 to 70). There were no differences in fatigue scores between genders (*P *= 0.99). Pain was also mainly scored as moderate (median = 6; IR 3.5 to 7). Again there was no statistical difference in pain scores between genders (*P *= 0.06). Significant differences were observed in fatigue scores between the Canadian and CDC-1994 groups, those in the former group having a median fatigue score of 70 (IR = 50 to 75), while in the CDC-1994 group the median fatigue score was 50 (IR = 45 to 70), *P *= 0.000.

The Canadian group had higher pain scores than the CDC-1994 group (respective medians were 6 (IR = 5 to 8) and 5 (IR = 3 to 7); *P *= 0.003). The group classified as non-cases had lower fatigue and pain scores (respective medians, 40 (IR = 40 to 60) and 2 (IR = 0 to 6) and in relation to the CDC-1994 group (*P *< 0.001).

## Discussion

This study investigated the epidemiology of ME/CFS in primary care in parts of East Anglia, London and East Yorkshire in England, using three case definitions. Primary care services in the UK can be used as an adequate setting for population based studies, due to their virtually universal population coverage [26-28]. While we do not claim to have systematically sampled communities to ensure UK representativeness, these three regions between them manifest most of the community types found in England [22,23,25].

The overall prevalence rate, for all case definitions combined, was 0.2%. Nevertheless, the population prevalence rate may be higher than that, as some people may not consult their GPs, or do not receive a diagnosis. Most cases conformed to the CDC-1994 definition, for which the prevalence was 0.19%, while about half (prevalence 0.11%) conformed to the Canadian definition. All but one of the cases who met the Canadian definition also met the CDC-1994 criteria, which suggests that the former may represent a particular subgroup of the latter. Rates according to the ECD were much lower, suggesting this case definition does not have sufficient diagnostic sensitivity to be useful on its own and we were therefore unable to validate it. Symptom prevalence and severity of cases, as indicated by the pain and fatigue scales were greater among those conforming to the Canadian definition than among those who did not, further enhancing the proposition that these patients may represent a distinct sub-group among those conforming to the CDC-1994 criteria. We have also compared quality of life of those meeting each of these case definitions, showing those meeting the Canadian criteria presented in general poorer results [29].

The overall prevalence rate of 0.2% in this study, or of 0.19% when only CDC-1994 criteria was used, is in the lower range of the commonly assumed population prevalence for the UK [6]. In general, these prevalence rates are also lower than those previously reported in primary care settings, such as 2.6% (or 0.5% when psychological morbidity was excluded) from a study carried out in the UK [30]; and 0.3% to 1.6% shown in studies from other countries [31-34], where the CDC-1994 was used. However, the rate we found is higher than in other studies based on case reports in the UK [35], Netherlands [36] and the US [37], which may have been subject to considerable under-reporting. This prevalence is similar to that of a seminal community based study carried out in the US of 0.24% [38], which also used a robust methodology for case detection. It is slightly lower than the prevalence found in other community based studies carried out in the US - 0.42% [39] and in Nigeria - 0.68% [40], which used similar methodologies. Studies using more inclusive diagnostic criteria have yielded prevalence rates up to ten times higher, for example 2.5% [41], and 1% [42], although potential for misclassification was higher, with some people with other fatiguing conditions perhaps being wrongly included. Bierl et al [41] studied CFS-like syndromes, and did not include clinical evaluation of patients.

Annual incident risks also varied among and within the three regions, with an overall risk of 0.15‰. Other estimates of incidence risk in the UK range from 0.05‰ [14] to 0.37‰ [43], and in the US, 0.18‰ [38]. Although we studied a large population, our findings, particularly for incidence estimates, should be interpreted with caution, due to the small number of cases and wide confidence intervals. It should also be noted that these are cases presenting for the first time with fatigue lasting for at least six months, but possibly following longer preceding periods of illness, due to possible delays in diagnosis; so, strictly speaking, they are not incident cases but rather new diagnoses of prevalent cases.

Possible reasons for the variation in results between studies include methodological differences, the exclusion, in our study, of potential cases which did not meet strict case definitions, under-diagnosis of cases, due to either lack of disease recognition or misconceptions about it by health professionals who may not accurately distinguish ME/CFS from other conditions presenting with chronic fatigue, or limited access to services.

Initial GP diagnoses may not always be accurate, and in our study 11% of those who had been labelled with one of the primary diagnoses were, on review, found by their GPs not to be cases, while 24% of GP-diagnosed cases did not fulfil the study case definitions. GP diagnoses alone would have generated a prevalence rate of 0.33%, reduced to 0.29% following GP case review. Reasons why GPs might not diagnose ME/CFS cases may include limited knowledge, or inability or unwillingness to recognise ME/CFS as a genuine disorder; lack of access of patients with ME/CFS to their GPs, due to symptom severity, disillusionment with health services and low expectations of finding help [15], or cultural reasons.

We used a robust methodology that involved the employment of specific diagnostic criteria and rigorously sequenced filter procedures before confirming cases. To maximize the identification of cases by GPs, in contrast to previous studies with primary care patients, we assessed recruited patients with unexplained chronic fatigue as well as those who had been labelled with a range of diagnoses that indicated they might be cases of ME/CFS. Failure to recognize cases was therefore much less likely in this study, except perhaps for individuals from ethnic minorities (see later), particularly as we worked with selected practices where the GPs were experienced in diagnosing and less likely to be unsympathetic to ME/CFS. While this may have compromised the representativeness of the study, we believe that working with non-engaged health professionals unwilling or unable to diagnose ME/CFS would have been more disadvantageous.

Despite the lower disease frequency we found compared to some other studies [30-34,39-41], the consequences for health and social care are still considerable, given the disabling nature of ME/CFS and the high economic impact for patients, families and the wider society [15,44,45]. Moreover the use of a large study population with a relatively wide geographical distribution enhanced the study validity.

Our findings of a higher risk of ME/CFS in women in all regions confirm previous findings [14,39,41,46]. The majority of our cases were reportedly white British, which contradicts previous studies that found similar or even higher prevalence rates in primary care among non-white groups [32-34,39-41]. It may be that ME/CFS is less commonly diagnosed among ethnic minority patients in UK primary care, and this under-diagnosis may well have contributed to the relatively low prevalence rate. A corollary of this may be the report by Haines *et al *of a higher prevalence of severe fatigue in adolescent girls from practices in less deprived areas, and their suggestion that this could reflect consulting behaviours [35]. Ethnicity would not, however, explain the higher rates in London, which are more likely due to consulting behaviours or disease recognition in densely populated urban areas. In addition, it is worth noting the relatively small number of cases used for inferences, particularly in London.

The cases recruited for this study had been ill for varying periods of time, but their fatigue and pain scores were still moderate to severe in most cases. Pain and fatigue scores were significantly lower in those chronically fatigued patients assessed as non-cases than in those who conformed to CDC-1994 or Canadian criteria, while the highest scores were found among those meeting this latter case definition. While symptom reporting is necessarily subjective, there was nevertheless a consistent and significant variation in the proportions of symptoms reported in these three groups. This suggests not only a distinction between ME/CFS and other chronically fatigued cases, but also that there is a distinction between ME/CFS patients who conform to the CDC-1994 alone, and those who conform also to the Canadian criteria. This is in line with the findings of Jason *et al *[5] and also reflect the stringency of the Canadian definition, in for instance requiring post-exertional fatigue. However, we have not found a higher frequency of GP diagnosis of fibromyalgia in Canadian positive cases (data not shown, P = 0.9), compared to CDC 1994. It is unclear whether cases conforming to the Canadian criteria represent the severe end of a disease spectrum, or if they represent a distinct clinical entity with distinct aetiology.

There is some evidence which indicates differences between ME/CFS and other chronically fatigued cases in relation to the type and severity of fatigue, somatic symptoms and biological abnormalities [47]. In the absence, at present, of reliable biomarkers, the choice of the most stringent available diagnostic criteria for ME/CFS for clinical and epidemiological studies is justified for its ability to distinguish true cases from non-cases, so as to enable reliable epidemiological inferences and to ensure study validity [48]. However, the ideal would be to sub-group research cases according to clinical and laboratory features [5,47,49-51] that may reflect different aetiologies and pathophysiologies. Use of the CDC-1994 definition in research is desirable as it has been widely used and has shown in our study to capture effectively nearly all cases studied. The improvement in criteria to incorporate more objective measures of severity and functional status, as suggested by Reeves [41], is desirable, and should reflect the true levels of limitation experienced by those with ME/CFS, to avoid any artificial inflation of prevalence statistics [52] and so encourage trivialization of the impact of such a severe disease on those who live with it.

## Conclusions

The difficulties in measuring the occurrence of ME/CFS reflect the absence of biomarkers for diagnosis and of specific, sensitive and reproducible clinical diagnostic criteria, and the variable perceptions and attitudes of health professionals about the illness with consequent inconsistencies in how they establish the diagnosis. We tried to minimize these difficulties by working with health professionals with a good understanding of the disease, by using a robust methodology based on a combination of clinical assessment and patient questionnaires, and by restricting diagnosis confirmation to those meeting strict study case definitions and appropriate exclusion criteria.

Our study yielded a minimum prevalence in primary care of 0.2% and a minimum incidence risk of 0.015%. Due to its disabling and long-term nature, and because it often affects individuals in the most productive parts of their lives, this represents a considerable burden to people affected and to society in general. However, it is important to recognize that frequency estimates vary according to how disease status or caseness is defined, and that the true burden of chronically fatiguing conditions is far higher, with the health needs for those with non-ME/CFS chronic fatigue also requiring attention.

To achieve optimum care for these heterogeneous groups of patients, it is very important that specific and discriminating case definitions are used, and that appropriate sub-grouping of cases is applied. This is at least as important for research, as the standard use of reliable and specific case definitions is essential to avoid differential misclassification bias when investigating associations and making causal inferences. This is vital to advance the understanding of mechanisms of diseases, the discovery of biomarkers, and development of specific treatments for those affected.

## List of abbreviations

BEM: Black and Ethnic Minorities; FMS: fibromyalgia; FS: fatigue syndrome; GP: general practitioner; IMD: Index of Multiple Deprivations; ME/CFS: myalgic encephalomyelitis/chronic fatigue syndrome; PIE: post-infectious encephalitis; PVAS: post-viral asthenic syndrome.

## Competing interests

The authors declare that they have no competing interests.

## Authors' contributions

LN and DP conceived the study and were its principal investigators. LN and EL analyzed and interpreted the data and wrote the manuscript. All authors contributed to data collection, interpretation of findings and review of the manuscript.

## Pre-publication history

The pre-publication history for this paper can be accessed here:

http://www.biomedcentral.com/1741-7015/9/91/prepub
